# Assessing the Effectiveness of Granular and Cell Suspension PGPR Biofertilizers in Enhancing Rice Growth in Saline Soils

**DOI:** 10.4014/jmb.2502.02008

**Published:** 2025-05-28

**Authors:** Pornrapee Sarin, Piyada Theerakulpisut, Surasak Siripornadulsil, Nuntavun Riddech

**Affiliations:** 1 Department of Microbiology, Faculty of Science, Khon Kaen University, Thailand; 2 Salt-tolerant Rice Research Group, Faculty of Science, Khon Kaen University, Khon Kaen 40002, Thailand

**Keywords:** PGPR, salt-tolerant rhizobacteria, biofertilizer, storage temperature, granular, cell suspension

## Abstract

In agriculture, plant growth-promoting rhizobacteria (PGPR) plays an important role in increasing soil quality and enhancing plant growth. However, the relative efficacy of various PGPR delivery modalities, such as granules and microbial suspensions, is uncertain. The aim of this study was to compare the efficiency of 12 salt-tolerant PGPR isolates as biofertilizers in two different formulations to determine which better supports rice seedling growth and microbial persistence under salinity stress. The selected isolates were tested for essential plant growth-promoting properties such as IAA synthesis, nitrogen fixation, phosphate and potassium solubility, ACC-deaminase activity, siderophore generation, and biofilm formation. Among the isolates, *Mesorhizobium* sp. S1-7 showed the highest IAA production at 74.43 μg/ml. Microscopic analysis confirmed that *Enterobacter aerogenes* P8, *Shinella* sp. R18, and *Mesorhizobium* sp. S1-7 successfully colonized rice roots under varying salinity levels (0, 2, 4, and 8 dS/m). In a pot experiment, both formulations increased rice seedling development in normal and saline environments. The liquid form exhibited greater stability during storage, whereas the granular form discharged more microbial cells into the soil. Despite their similar impacts on plant growth, the granular form has a significant advantage due to its slow-release qualities, which promote microbial persistence and long-term advantages in tough settings like saline soils. This study highlighted the potential of *Enterobacter* sp., *Shinella* sp., and *Mesorhizobium* sp. in granular biofertilizer formulations and contributed to developing optimized biofertilizer strategies for enhancing crop productivity in salinity-affected regions.

## Introduction

Abiotic stresses, particularly salinity, are prevalent in various environments, significantly impacting plants, animals, and humans. Saline soil, commonly found in many countries, pose a severe threat to crop growth and productivity due to the accumulation of salt ions, which can inhibit photosynthesis and nutrient uptake in plants [1]. The consequences of salt, combined with methods of irrigation and water evaporation, lead to decreased soil structure, reduced organic matter decomposition, and limited nutrient mineralization. Microbial by-products, such as exopolysaccharides and biofilms, provide potential solutions by improving soil aggregation and increasing water retention capacity [2].

The rhizosphere, a complex zone of interaction between soil, water, plant roots, and microbes, is critical in addressing these difficulties. Plant growth-promoting rhizobacteria (PGPR) that naturally live in the rhizosphere have been recognized for their capacity to increase root health, facilitate nutrient uptake, and improve plant resilience to abiotic stressors [3, 4]. PGPR promotes plant growth by fixing nitrogen, solubilizing phosphorus and potassium, producing siderophores, and synthesizing phytohormones [5]. Under salinity stress, halotolerant PGPR produces unique features such as osmoprotectants (*e.g.*, proline, trehalose) and 1-aminocyclopropane-1-carboxylate (ACC) deaminase, which assist manage osmotic stress and lower ethylene levels, promoting plant growth [6]. Numerous researchers have highlighted the role of PGPRs in enhancing salt tolerance in various plants. For instance, *Arthrobacter* sp. improved wheat and pea growth by stimulating nutrient uptake of plants [7], *Enterobacter* sp. reduced ethylene production in *Arabidopsis thaliana* plant [8], *Klebsiella* sp. increased K^+^ and proline content in oat plant [9]. *Pantoea* sp. aided mung bean plants in salt tolerance through ACC deaminase activity [10]. Moreover, several previous reports have indicated that bacteria that are associated with plants belong to different genus such as *Microbacterium*, *Rhizobium*, *Pseudomonas*, *Bacillus*, *Paenibacillus*, *Burkholderia*, *Methylobacterium*, and *Azospirillum*, etc. have also been recognized for their ability to enhance plant resilience under abiotic stress conditions [11, 12]. However, most research focuses on their physiological mechanisms rather than optimizing biofertilizer formulations for practical application.

Biofertilizers, which are commonly manufactured as liquid suspensions or immobilized granular forms, are gaining popularity due to their potential to increase crop productivity in a sustainable manner. Liquid biofertilizers are noted for their quick action and long shelf life, but granular formulations, particularly those containing carriers like sodium alginate, allow continuous microbial release and improved soil characteristics [13]. Sodium alginate, an environmentally acceptable polymer, has emerged as an excellent carrier for PGPR due to its hydrogel-forming capabilities, biocompatibility, and inexpensive manufacture [14]. Despite the increased interest in PGPR-based biofertilizers, little study has been conducted to compare the efficacy of certain PGPR species, such as *Enterobacter* sp., *Shinella* sp., and *Mesorhizobium* sp., in various formulations under salinity stress. Although no single formulation is universally optimal due to the unique advantages and limitations of each type [15], carrier design plays a critical role in maintaining PGPR viability and efficacy.

Understanding whether liquid suspensions or sodium alginate-based granules are more effective in preserving microbial viability and promoting plant growth is vital for developing targeted biofertilizer applications. This study bridges a knowledge gap by evaluating the performance of *Enterobacter* sp., *Shinella* sp., and *Mesorhizobium* sp. in suspension and granular biofertilizer forms under both normal and saline soil conditions. The primary objective was to determine which formulation better supports rice seedling growth and microbial persistence under salinity stress, providing actionable insights for designing effective biofertilizers tailored to challenging environments. These findings will contribute to the development of more efficient biofertilizer strategies for enhancing sustainable agriculture in salinity-affected regions.

## Material and Methods

### Isolation and Characterization of 12 Selected PGPR Isolates

Twelve isolates of microorganisms were screened from rhizosphere soil of rice (*Oryza sativa*), Jerusalem artichoke (*Helianthus tuberosus* L.) Kale (*Brassica oleracea var. Alboglabra*), and water spinach (*Ipomoea aquatica Forsk*.) in Muang Khon Kaen, Khon Kaen Province, Thailand (Latitude and longitude coordinates are: 16.439625, 102.828728). To prepare serial dilutions up to 1 × 10^8^ CFU ml^-1^, 100 μl aliquots from each dilution were spread on tryptic soy broth (TSB) agar and incubated for two days at 28 ± 2°C. Morphologically distinct bacterial colonies were chosen for further purification. The pure isolates were temporarily stored at -20°C in a 20% glycerol solution for future experiments.

### Testing of Plant Growth Promotion Activities of PGPR

**Indole 3-acetic acid content assay.** Twelve isolates were cultured in TSB supplemented with 1 g l^-1^ of L-tryptophan. The mixture was incubated in a shaking incubator at 30°C and 150 rpm in the dark condition for 2 days. Bacterial suspension was centrifuged at 8,000 rpm for 5 min, only 2 ml of supernatant was mixed with 2 ml of Salkowski’s reagent (1 ml 0.5 M FeCl_3_, 50 ml 35% HClO_4_). The pink color solution was shown after incubating at the dark condition for 20 min, then the solution was measured the indole 3-acetic acid content by using spectrophotometer (Thermo Fisher scientific, model Genesys 180, USA) at 535 nm and comparing with indole-3-acetic acid standard [16].

### Assay of Indole-3-Acetic Acid (IAA) Products on TLC Chromatography

IAA production was confirmed through thin-layer chromatography (TLC). Rhizobacteria were cultured in TSB supplemented with 1 g l^-1^ of L-tryptophan and incubated on a shaker at 150 rpm and 30°C for 48 h. Bacterial suspension was centrifuged, supernatant was adjusted an acidic pH(pH 2.0-3.0) and then the supernatant was mixed with ethyl acetate (1:1), then shaking for 10 min. Crude of IAA extract solution was analyzed by using TLC. The crude extract IAA and IAA standard (Indole-3-acetic acid, Acros Organics, USA) were spot on TLC silica gel 60 F_254_ (Merck, Germany). TLC plate was soaked into chamber which was contained butanol-ethyl acetate -ethanol - water (3:5:1:1) until the mobile phase reached on the top of TLC plate, TLC plate was dry at room temperature and after that sprayed with Salkowski’s reagent, the positive result of red spot was shown after TLC plate drying [17]. Spots with Rf values that coincided with those of legitimate IAA were discovered under UV light (254 nm) by spraying Ehmann reagent on TLC plates.

$$\text{Calculating the Rf value of a compound: Rf value}=\frac{\text{distance traveled by compound}}{\text{distance traveled by solvent front}}$$

**Enzyme activity.** Rhizobacterial was tested for nitrogen fixation activity on Ashby nitrogen-free agar plate [18]. Phosphate solubilization activity was performed on National Botanical Research Institute's phosphate growth medium (NBRIP) agar which supplemented with tricalcium phosphate and Potassium solubilization activity was detected on Aleksandrov agar supplemented with 2 g l^-1^ of potassium aluminum silicate, the results were examined by observing the clear zone forming around colonies on agar plates [19]. Diameter of colony and diameter of clear zone were measured and calculated for phosphate and potassium solubilizing index (PSI) following the formula:

$$\text{PSI}=\frac{\text{Diameter of clear zone}}{\text{Diameter of colonyt}}$$

[20].

Siderophore production activity was assayed on chrome azurol agar, positive result was showed the yellow, orange and purple zone around the colony that indicated siderophore production [21].

**ACC deaminase activity.** Rhizobacteria were cultured in TSB and incubated at 30°C with 150 rpm for 24 h. Bacterial suspension was centrifuged, and cell pellet was washed with 0.85% NaCl and sterile distilled water for twice, respectively. Cell pellet was re-suspended with 0.1 M Tris–HCl (pH 7.5). Bacterial suspension was point inoculated on DF (Dworkin and Foster) minimal medium (DF salts per liter: 4.0 g KH_2_PO_4_, 6.0 g Na_2_HPO_4_, 0.2 g MgSO_4_·7H_2_O, 2.0 g glucose, 2.0 g gluconic acid and 2.0 g citric acid with trace elements: 1 mg FeSO_4_·7H_2_O, 10 mg H_3_BO_3_, 11.19 mg MnSO_4_·H_2_O, 124.6 mg ZnSO_4_·7H_2_O, 78.22 mg CuSO_4_·5H_2_O, 10 mg MoO_3_, pH 7.2) that varied nitrogen source which included (NH_4_)_2_SO_4_ (positive control) [22], 3 mM 1-Aminocyclopropane-1-carboxylic acid or ACC of rhizobacteria on DF medium which supplemented with ACC [23].

**Bio-film production.** Rhizobacteria were inoculated into TSB in polystyrene tube, incubated at 30°C, for 48 h under static condition. Biofilm production activity was started with taking out the culture broth and washed with sterile distilled water 3 times. Next step, 0.1% crystal violet was added in to polystyrene tube, incubated for 30 min, then crystal violet was poured out and washed with 70% ethanol. If bacteria produced biofilm, the purple film appeared on the surface of polystyrene tube [24].

### Root Colonization Evaluation Assay

Rhizobacterial suspension was adjusted to 10^8^ CFU/ml. The mixture-inoculum (three isolates of rhizobacterial) was mixed with ratio 1:1:1. Rice seeds were sterilized with 70% Alcohol for 5 min and 6% sodium hypochloride for 10 min respectively. Finally, seeds were washed with sterile distilled water three times. The sterile rice seed was soaked in bacterial suspension for 30 min, then five seeds were transferred into the bottom of Hoagland’s solution that adjusted the salinity levels with NaCl solution from 0%, 0.25%, to 0.5 % NaCl. The bottom was kept in a dark condition and incubated at room temperature for 2 weeks. The germ seed was sampled to study the interaction of root colonization between microbes and root of rice by scanning electron microscope and spread plate technique.

**Scanning electron microscopy (SEM).** Rice seedling was separated into the root and shoot parts after 14 days of cultivation, root was washed with 0.1 M phosphate buffer pH 7.0 for twice. Next step, root was fixed with 2.5%glutaraldehyde at 4°C for 2 h., then root washed with 0.1 M phosphate buffer pH 7.0 for three times. Root samples were dehydrated by incubation in a gradual of absolute ethanol solutions (Absolute ethanol concentration at 50%to100% respectively), solvent concentration was increased gradually, thus, water was removed. Root sample was dried by using the critical point drying (CPD), Polaron Range, model CPD 7501. The samples were placed on aluminum stubs that double coated with carbon conductive tape and sputtered with gold (Sputter coater machine of Cressington, model 108auto). Observation of root colonization was performed under the scanning electron microscope (SEM) (LEO, model 1450VP, England) with BE VPSE detector.

**Root colonization assay by conventional method.** One g of germinated rice root (14 days old) was soaked in the diluent of 0.85% NaCl solution under shaking condition at 150 rpm for 30 min. The solution was diluted and spread on tryptic soy agar (TSA) and incubated at 30°C for 24-48 h. Total amount of rhizobacteria that colonized the rice root was quantified by counting the colonies form on agar plate and calculated the colony forming unit (CFU/g).

### Biofertilizer Production

**Granulated form.** A mixture of PGPR isolates (1:1:1 v/v/v) was immobilized on alginate carriers supplemented with skim milk and sterilized before granule formation using CaCl_2_ solution (which was prepared by weighting 9.11 g of CaCl_2_ and dissolved this chemical in 500 ml of sterile distilled water), then let the bio-fertilizer granule was soaked on this solution for 20 min. Granules were stored for further analysis.

**Liquid form.** Suspensions of PGPR isolates were prepared in 0.85% saline solution with a concentration of 10^8^ CFU/ml and used directly as liquid biofertilizer.

### Survival and Viability of Rhizobacteria

Rhizobacterial in the form cell suspension and cell immobilized on sodium alginate granule were prepared and studied on shelf life during the incubation at room temperature and 4°C in sterile saline and normal soils for 1 month. The survival cells of microbial inoculants were counted on TSA agar plate by spread plate technique and then calculated the log number of survival cell at 7, 14, 21 and 30 days.

### Releasing of Rhizobacteria Number from Biofertilizer

This test was performed by inoculating 10% (W/W) the granule bio-fertilizer and rhizobacterial suspension forms into 200 g of sterile normal soil and saline soil (normal soil that was supplemented with 0.5% NaCl solution) which contained in tissue culture bottle size 24 oz. The releasing of rhizobacteria isolates from two kinds of biofertilizer on day 7, 14, 21 and 30 of incubation in soils was counted on TSA agar by spread plate technique. The colony forming unit per gram of soil was calculated.

### Measured Responses

**Effect of biofertilizer on the growth traits of rice seedling in saline and normal soil.** Three kg of soil were prepared in a plastic pot, rice seedling 7-day-old were transferred into pot under two types of soil conditions as follow; saline soil (normal soil supplemented with 0.5% NaCl) and normal soil (natural soil), two L of water were added into the pot. After that, bio-fertilizers were added into pot following the 3 treatments: control (without fertilizer), granule of rhizobacterial immobilized on the alginate, commercial liquid organic fertilizer (EM Kyusei, Thailand) and rhizobacterial suspension. This experimental design was a complete random design (CRD). Rice was grown for 30 days, plants analyzed the shoot length, root length, shoot and root dry weight, total biomass, electrical conductivity (EC), pH, fluorescein diacetate hydrolysis enzyme (FDA).

**Chlorophyll content in plants.** A total of 0.05 g of rice leaves were added into test tube which containing 5 ml of 80% acetone and incubated at room temperature in the dark condition for 48 h. The sample was measured by using spectrophotometer at 645 and 663 nm, the value was calculated chlorophyll content following the method of Arnon [25].

**Proline content in plants.** A total of 0.5 g of fresh weight of plant were extracted with 10 ml of 3% Sulfosalicylic acid, the sample was thoroughly mashed and filtered through the filter paper. Two ml of extracted solution were mixed with 2 ml of acid ninhydrin and 2 ml of glacial acetic acid, then the sample was boiled at 100°C for 1 h. Reaction was stopped in the ice bath. Next step, toluene was added and mixed all of ingredients, the sample was stand for separating the layer of solution. The toluene layer was on the top and measured by using a spectrophotometer at 520 nm and the proline content was calculated and compared with standard [26].

**Soil properties.** The total enzyme activity of microorganisms in the soil was analyzed by fluorescein diacetate hydrolysis (FDA). 7.5 ml of 60 mM potassium phosphate buffer pH 7.6 and 0.1 ml of FDA stock (1,000 mg) were added into one g of soil sample (moisture content was approximately 40-50%), The sample was incubated on shaker incubator, 150 rpm at 30°C for 30 min, after that 7.5 ml of chloroform: methanol (ratio 2:1) were mixed with the solution. afterward, samples were centrifuged at 8,000 rpm for 10 min, the solution on top was measured the total enzyme activity by the spectrophotometer at 490 nm that compared with standard FDA (Fluorescein diacetate, Acros Organics, USA) [27].

### Molecular Identification

Selected PGPR isolates were identified through 16S rRNA gene sequencing. Culture each of bacterial in Nutrient broth for 24 h. added into the tube and sample was spin by using centrifuge at 10,000 rpm for 2 min, then discarded the clear solution. DNA extraction was extracted using TIANamp Bacteria DNA Kit (TIANGEN, Chaina). DNA sample was analyzed with 1.0% agarose gel. The 16s rRNA genes were amplified by using the extracted DNA as a template in the PCR reaction. The primers used in this study were 8F primer (5-AGA GTT TGA TCM TGG CTC AG-3) [28] and 1512R primer (5-ACG GYT ACC TTG TTA CGA CTT-3) [29]. PCR product was detected the DNA band by using gel electrophoresis. Next, PCR products were purified and submitted to ATCG company, Thailand for sequencing. The DNA sequences were analyzed against Genbank databases from the National Center for Biotechnology Information (NCBI) server.

### Statistical Analysis

All the data was analyzed by one-way ANOVA analysis of variance and performed using Statistic 10 program and compared the data with Fisher's Least Significant Difference (LSD) method.

## Results

### Production of IAA

**Fig. 1:**
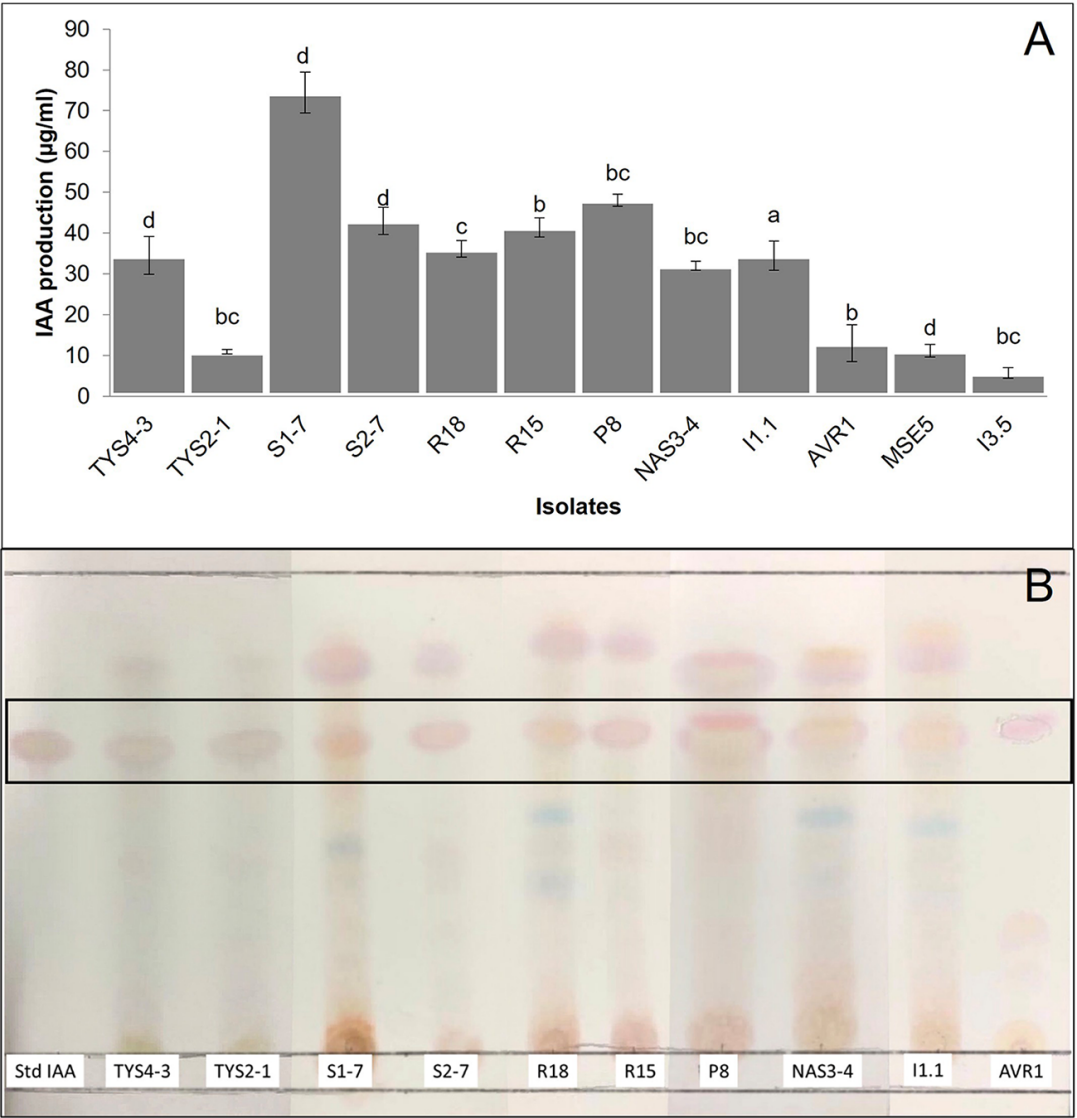
Indole-3-acetic acid (IAA) produced by 12 rhizobacterial isolates. (**A**) Quantification of IAA production (μg/ml) by each isolate using a colorimetric assay. Different letters indicate statistically significant differences at a 95% confidence level (*p* < 0.05) based on the LSD test. (**B**) Thin-layer chromatography (TLC) analysis of IAA production, where the intensity of pink bands represents the presence of IAA, compared to the standard (Std IAA).

IAA producing bacteria was used to screen for the multifunctional activities of plant growth promotion. The measurement of IAA production among all 12 isolates cultured in the tryptic soya broth supplemented with tryptophan 1,000 mg l^-1^ showed that isolate S1-7 produced the highest hormone production, reaching a level of 74.43 μg/ml and revealed the statistically significantly different compared to other treatments (Fig. 1A).

### TLC Chromatography Assay of IAA Production

This test was established to verify the IAA production capability of 12 rhizobacterial isolates using TLC chromatography. It was found that IAA standard showed pink spot-on TLC plate and presented Rf value as 0.68. Among 12 rhizobacterial results, 10 isolates displayed pink spots, including isolate TYS4-3, TYS2-1. S1-7, S2-7. R18, R15, P8, NAS3-4, I1.1 and AVR1, demonstrating Rf values in the range of approximately 0.67-0.69, closely resembling the IAA standard (Fig. 1B).

### Functional Characterization of Plant Growth-Promoting Rhizobacteria (PGPR) Isolates

**Fig. 2:**
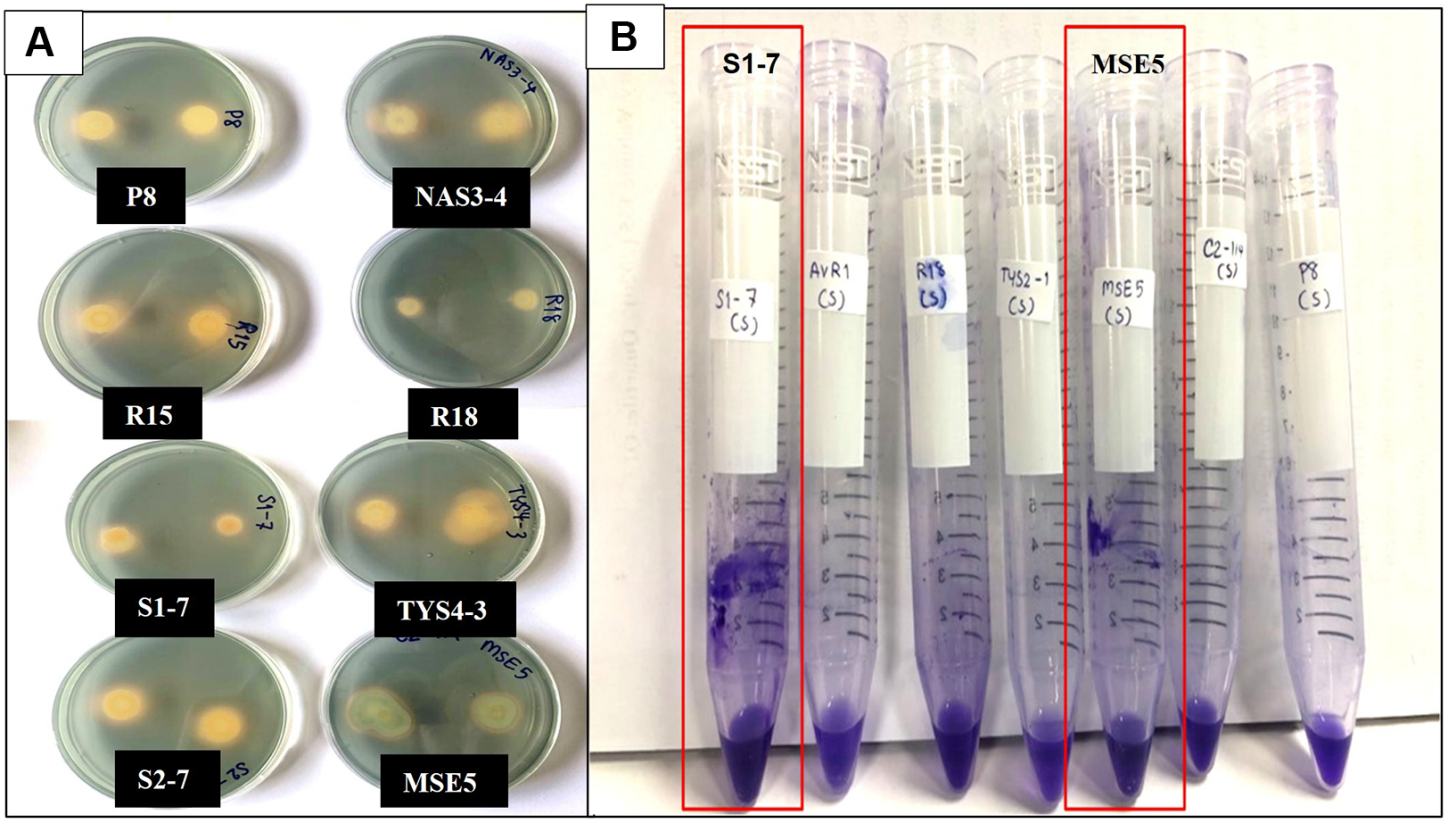
A: Siderophore production by 8 isolates (isolate NAS3-4, R18, TYS4-3, MSE5, P8, R15, S1-7, S2-7) demonstrated through the formation of halos around bacterial colonies on Chrome Azurol S (CAS) agar. B: Biofilm Production by Isolates S1-7 and MSE5. Positive Results Indicated by a Ring of Purple Color Around the Polystyrene Tubes.

**Fig. 3:**
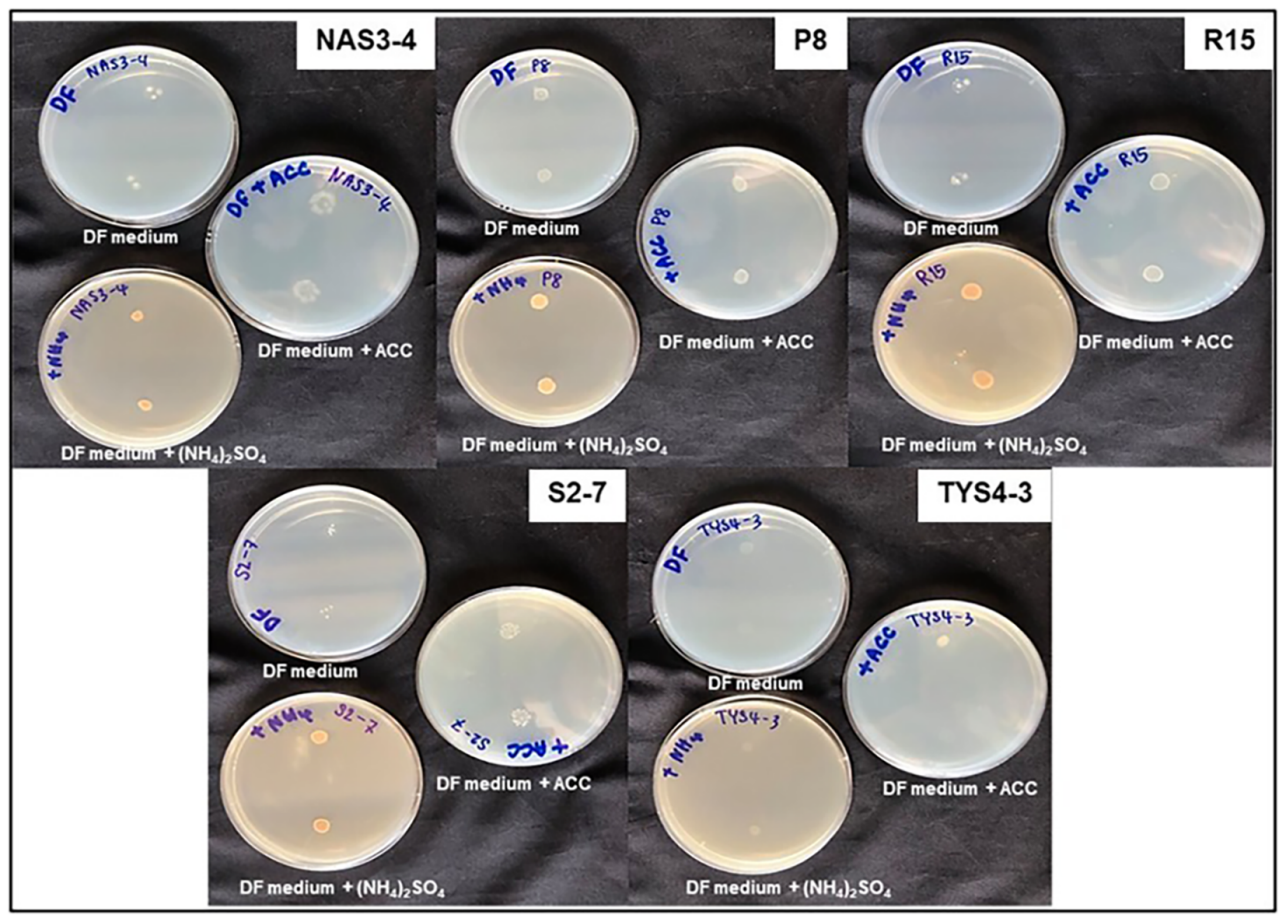
1-aminocyclopropane-1-carboxylate (ACC) deminase activity by 5 isolates (isolate NAS3-4, P8, R15, S2-7, TYS4-3) assessed on three types of DF minimal agar media: DF medium without nitrogen source, DF medium supplemented with ACC, and DF medium supplemented with (NH_4_)_2_SO_4_.

For the nitrogen fixation activity, it was performed on nitrogen free medium (ashby’s agar), all 12 isolates exhibited a positive result by forming colonies on the medium. This indicated that the rhizobacteria were able to fix the nitrogen gas from the air. Phosphate solubilization was observed on the national botanical research institute's phosphate growth medium (NBRIP) which added tricalcium phosphate (Ca_3_(PO_4_)_2_). Only two isolates (P8 and R18) showed a clear zone around colonies, indicating their ability to solubilize tricalcium phosphates as a phosphate substrate in the medium. Potassium solubilizing was studied on Aleksandrov agar that supplemented with potassium aluminum silicate, only isolate P8 showed the capable of potassium solubilization activity. The siderophore production test releaved the orange colored clear zones on chrome azurol S agar, indicating that 8 isolates were capable of produce siderophores (Fig. 2A). Additionally, ACC deaminase activity test was performed on three kind of DF minimal agar, identifying 5 isolates that could degrade ACC instead of using nitrogen as a source (Fig. 3). Notably, ACC deaminase activity was observed under stress conditions, such as high salinity and drought stress in soil. Biofilm production was also tested, it was found that isolate MSE5 and S1-7 were showed the positive result (Fig. 2B). Isolate S1-7, P8 and R18 were showed high content of IAA production and also exhibited on the multifunctional activities. Thus, these three isolates were selected for further study (all of the data shown on Table 1)

### Molecular Identification of the Selected PGPR

The selected PGPR isolates' 16S rRNA gene segments were sequenced, aligned, and BLAST evaluated against bacterial sequences in the GenBank database (NCBI). The results revealed that isolate P8 exhibited 99.16%similarity to *Enterobacter aerogenes*, while isolates R18 and S1-7 showed high sequence homology to *Shinella* sp.(99.09%) and *Mesorhizobium* sp. (99.58%), respectively.

### Root Colonization at Different Salinity Levels

**Fig. 4:**
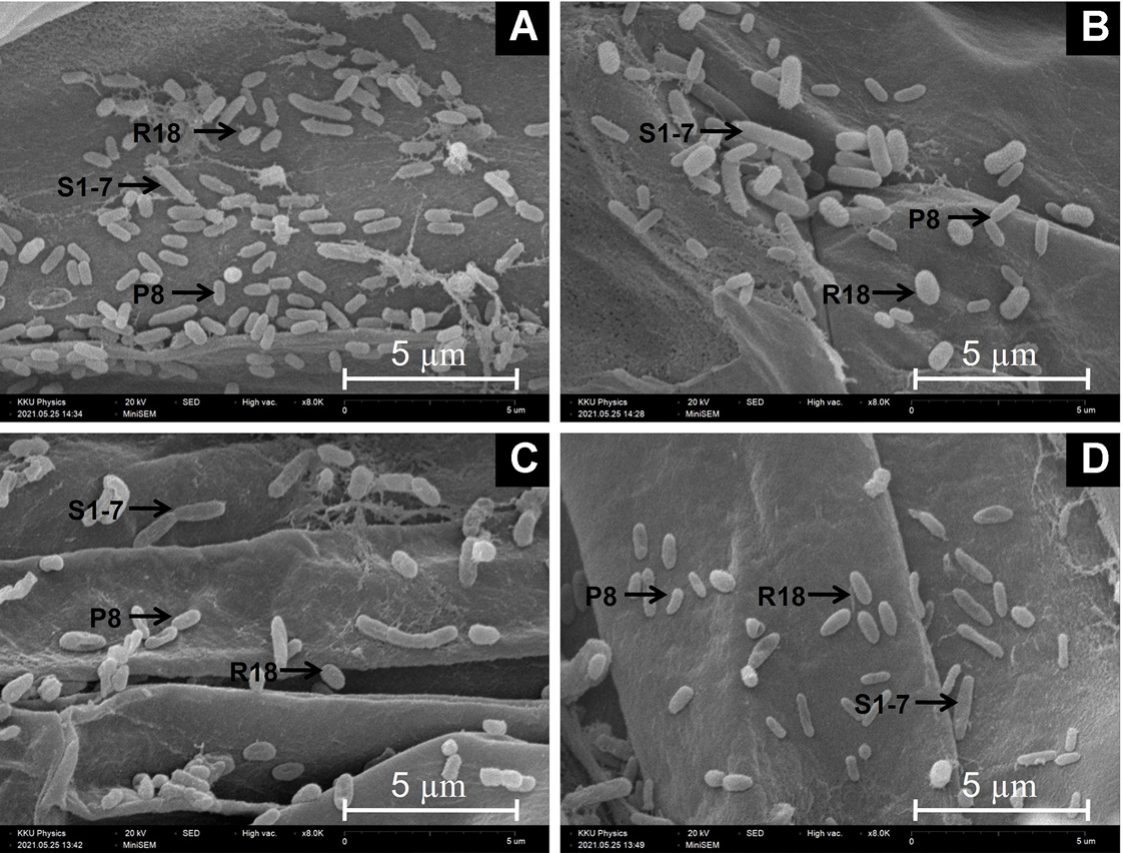
Showing of the colonization of the mixture of 3 isolates of rhizobacteria (*Enterobacter aerogenes* P8, *Shinella* sp. R18, and *Mesorhizobium* sp. S1-7) on root surface of rice seedling under 4 level of salinities by various the concentration of NaCl solution including salinity 0 dS/m, 2 dS/m (0.12 g NaCl), 4 dS/m (0.25 g NaCl) and 8 dS/m (0.50 g NaCl).

**Fig. 5:**
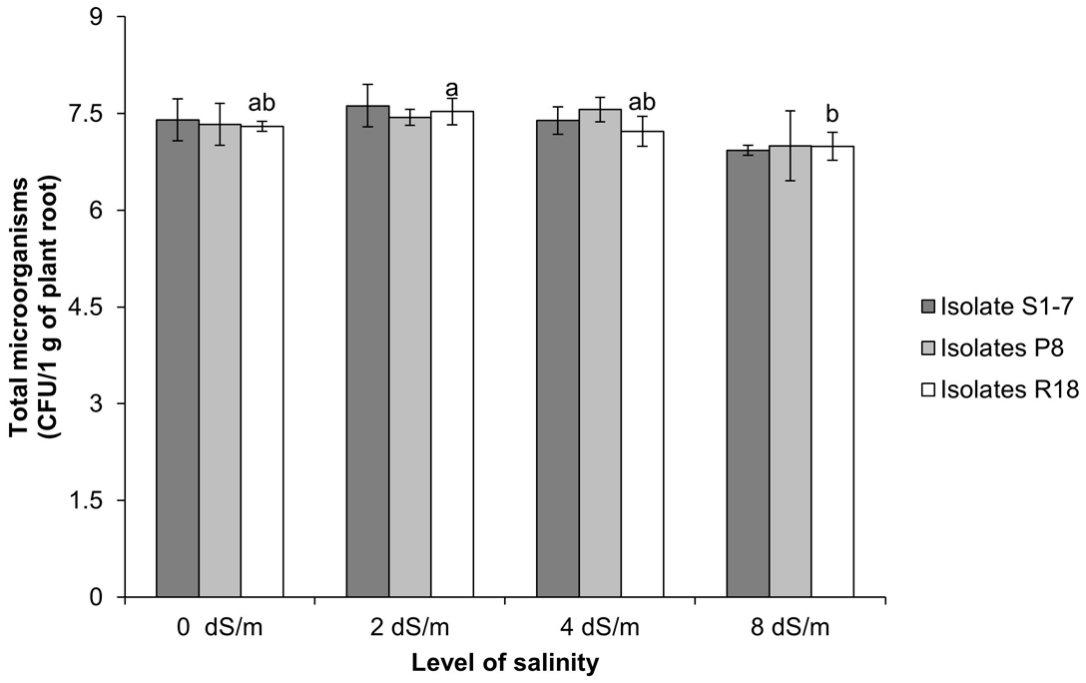
Total microorganisms of 3 isolates colonized on root surface of rice seedling under 4 salinities levels (salinity was varied using NaCl solution) that include salinity 0 dS/m, salinity 2 dS/m (0.12 g NaCl), salinity 4 dS/m (0.25 g NaCl) and salinity 8 dS/m (0.50 g NaCl) by spread plate technique observation. Different letters (a, b, ab) in the bar graph represented significant differences among treatments (*P* < 0.05) according to the LSD test.

The capacity of indole 3 acetic acid producing rhizobacteria (*E. aerogenes* P8, *Shinella* sp. R18, and *Mesorhizobium* sp. S1-7) tocolonize rice roots was evaluated across varying salinity levels using SEM, alongside quatification of viable cell densities through the spread plate technique. Rice seedlings at 14 days of age was used for this observation. The result found that all three rhizobacteria were capable of colonizing the root surface across 4 salinity level tested (0, 2, 4, 8 dS/m), as evidenced by SEM (Fig. 4). Moreover, for counting the total microorganisms that were colonized on root surface, the result showed that the density of isolate *Mesorhizobium* sp. S1-7 and *E. aerogenes* P8 were not different in all of conditions. Except for isolate *Shinella* sp. R18 that was decreased in number under salinity level 8 dS/m (Fig. 5)

### Effect of Temperature on Bacterial Viability in Granular and Liquid Biofertilizers

**Fig. 6:**
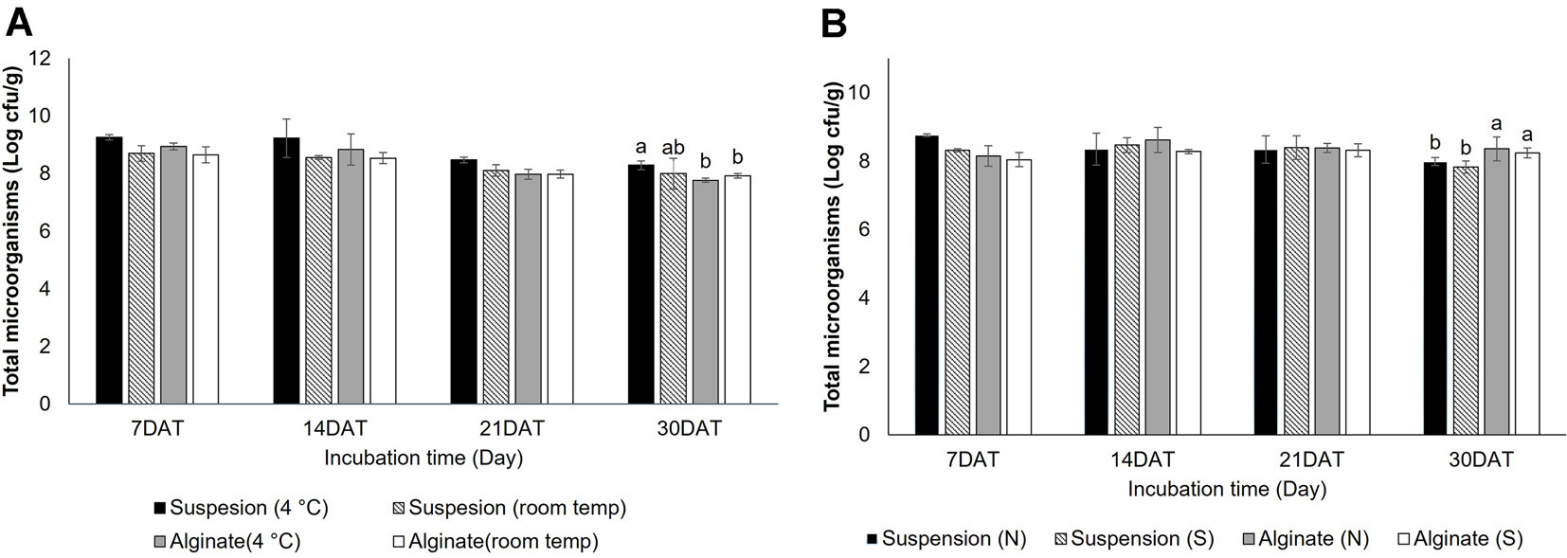
Survival and release of indole-3-acetic acid (IAA)-producing rhizobacteria in different formulations. (**A**) Bacterial survival in suspension and alginate-immobilized granules stored at different temperatures (4°C and room temperature) over time. (**B**) Release of microorganisms from both formulations in normal (N) and saline (S) soil conditions (adjusted to 0.5% NaCl). Different letters indicate statistically significant differences at *p* < 0.05.

To check the viability and eficacy of rhizobacteria in both granular and cell suspension form for 1 month storage period at both 4°C and room temperature for a month, the total number of survivals cells of rhizobacterial was counted on the agar plate. The cell density of the microbial inoculum on bio-fertilizer started at 10^9^ CFU/g. Two forms of bio-fertilizer were not significantly different at both temperatures except for 30 days, with the density of microbes in all of treatments decreased. However, bacterial suspension (with density 10^8^ CFU/g) which kept at 4°C was presented as the number of survival cells of microbe higher than it was in sodium alginate (with density 10^7^ CFU/g) form at both temperatures (Fig. 6A). Furthermore, the releasing of microbial cells from the granular bio-fertilizer compared with cell suspension was also tested. The result showed that the releasing of microbes was no significant difference at 7, 14, 21 and 30 days in soils. At the 30-day mark, the granular bio-fertilizer showcased a substantially higher microbial density of around 10^8^ CFU/g, surpassing the other treatments (density 10^7^ CFU/g)(Fig. 6B). These findings indicate that lower temperatures (4°C) significantly enhance bacterial survival, as metabolic activities slow down, reducing cellular stress and prolonging viability. In contrast, at room temperature, bacterial populations decline more rapidly due to nutrient depletion and increased metabolic activity.

### Effect of Granular Biofertilizer on Rice Growth under Saline Condition

**Fig. 7:**
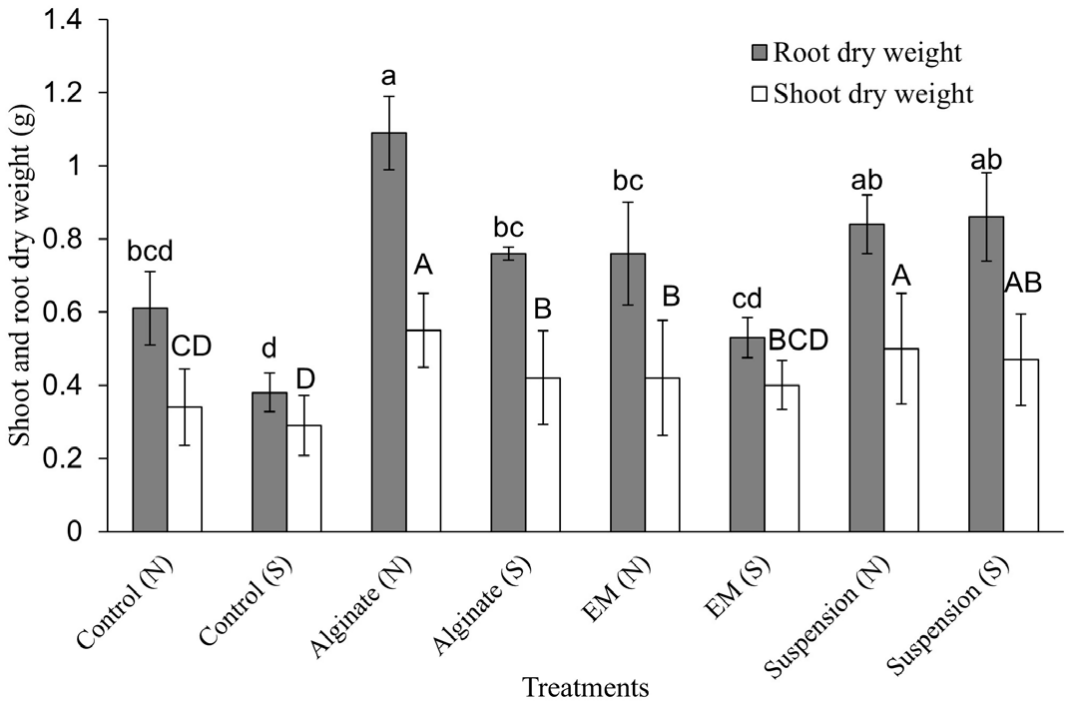
Effect of bio-fertilizer on shoot dry weight and root dry weight of rice at one month old and soil under the cultivation on normal soil(N) and saline soil (S/ normal soil was adjusted salinity with 0.5% NaCl (w/w)). Eight treatments as follows: control (without fertilizer), rhizobacterial immobilized on sodium alginate (granular biofertilizer), Effective microorganism (EM) and rhizobacterial suspension were applied into two types of soils in this experiment. Different letters in the bar graph represented significant differences among treatments (*p* < 0.05) according to the LSD test.

**Fig. 8:**
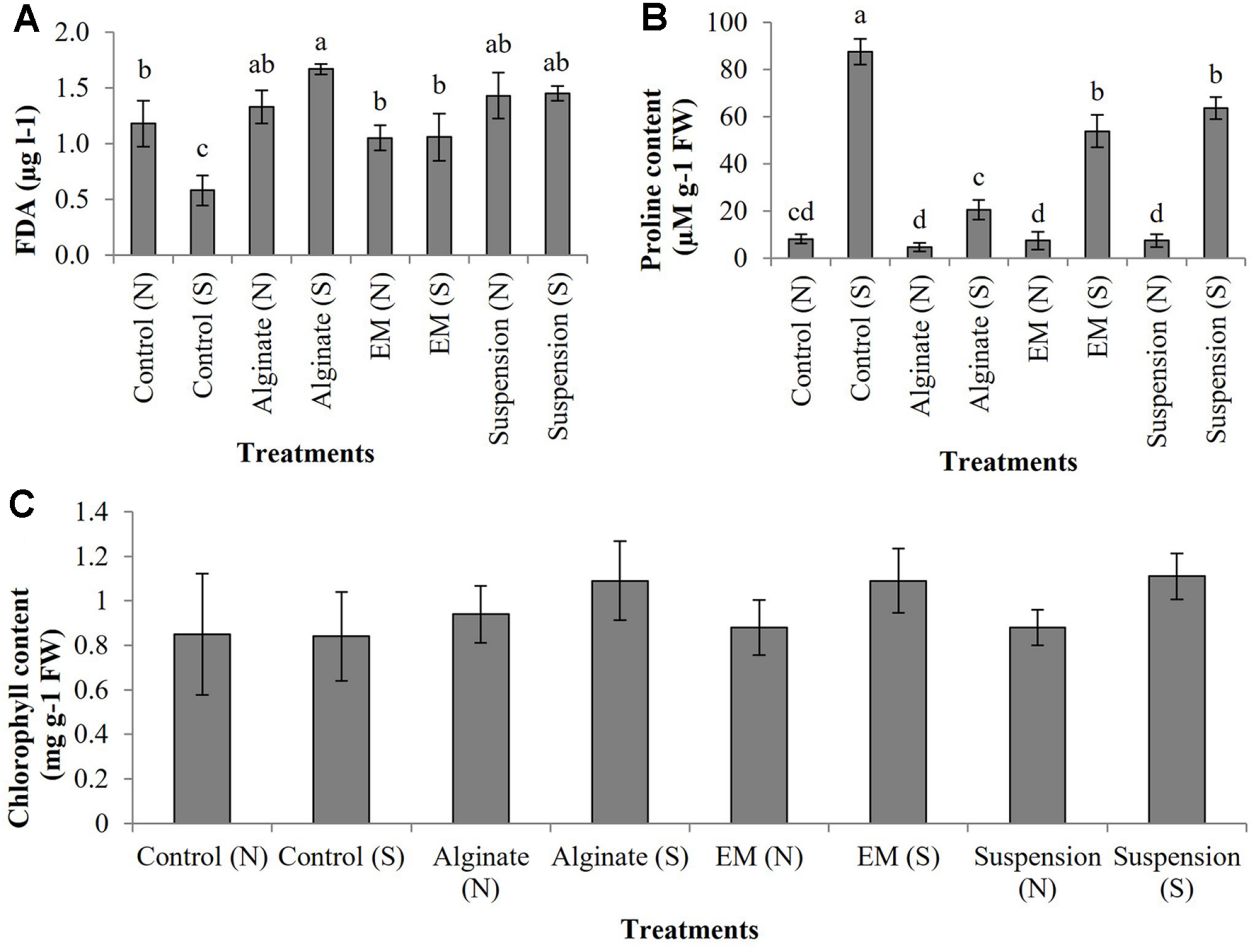
Effect of bio-fertilizer on the growth of rice and soil under the cultivation on normal soil(N) and saline soil (S/ normal soil was adjusted salinity with 0.5% NaCl (w/w)), (**A**) Measurement of fluorescein diacetate (FDA) activity in cultivated soil. (**B**) Proline content in plant leaves. (**C**) Chlorophyll content in rice leaves. Eight treatments as follows: control (without fertilizer), rhizobacterial immobilized on sodium alginate (granular bio-fertilizer), Effective microorganism (EM) and rhizobacterial suspension were applied into two types of soils in this experiment. Eight treatments as follows: control (without fertilizer), rhizobacterial immobilized on sodium alginate (granular bio-fertilizer), Effective microorganism (EM) and rhizobacterial suspension were applied into two types of soils in this experiment. Different letters in the bar graph represented significant differences among treatments (*p* < 0.05) according to the LSD test.

To assess the effectiveness of bio-fertilizer on the growth of rice seedling, two diferent forms of bio-fertilizer (rhizobacterial suspension and granular form in sodium alginate) were inoculated to stimulate rice growth in both saline (normal soil supplemented with 0.5% NaCl solution) and normal soil conditions. The results demonstrated significant enhancements in rice growth. Specifically, in normal soil, the application of bacterial suspension (T7) and granule form (T3) led to highly significant improvements in rice growth parameters. This was reflected in increased shoot length (51.8 and 49.8 cm), leaf count (4.5 and 4), and shoot and root dry weight (1.34 and 1.64 g of dry weight) (Fig. 7). In addition, under stress condition (in saline soil), the bacterial suspension (T8) could promote rice seedling growth better than rhizobacterial immobilized in sodium alginate (data shown on Table 2 and Fig. 7). Chlorophyll content in rice leaves was detected and it was found no significant difference in all treatments (Fig. 8C). Proline content in plant leaves was observed across all treatments in the saline soil, indicating that leaves accumulated higher proline content under stressful conditions compared to plants grown in normal soil. Notably, in the control treatment under saline soil, proline content accumulation reached 87.55 μM g^-1^ FW, representing a significantly higher increase compared to other treatments (Fig. 8B). For FDA activity in cultivated soil, all treatments that were inoculated with both forms (rhizobacterial suspension and granular bio-fertilizer), indicating a higher increase in an activity of FDA compared to the un-inoculated control (Fig. 8A). The pH and EC were detected in original and cultivated soils to evaluate the impact of biofertilizer application on soil chemical properties. No significant differences were noted in pH values between the two types of soil, which consistently ranged from 7.18 to 7.47. In normal soil, the EC value was shown to be an average in the range of 0.28-0.56 dS/m. In saline soil, where normal soil was adjusted salinity with 0.5% NaCl (w/w), the EC value were approximately in the range of 3.98-4.56 dS/m. The pH was also not shown differently from normal soil (Table 3). The pH results in harvested soil suggested that both forms of biofertilizer treatment did not strongly influence soil acidity or alkalinity, maintaining a neutral pH range suitable for plant growth. Meanwhile, the significantly higher EC in saline soil highlights the difficulty of salt stress, emphasizing the need to investigate whether biofertilizer treatments can improve plant tolerance under such conditions.

## Discussion

### The Symbiotic Relationship between Plants and PGPR

Plants engage in a symbiotic relationship with soil microorganisms such as bacteria and fungus, during their growth and development. Among these host-associated soil microorganismsare PGPR that exert beneficial effects on plants species and confer beneficial effects to host plants through direct and indirect mechanisms [30]. These mechanisms include nutrient uptake, increased nutrient availability by the mineralization of organic compounds, solubilization of minerals, and production of phytohormones [31]. These microbes are commonly referred to as PGPR, contributing to plant health in an environmentally eco-friendly manner [32]. In this study, twelve isolates of rhizobacteria were obtained from the rhizosphere of various plants, such as rice, Jerusalem artichoke (sunchoke), and vegetables. This study aimed to identify and evaluate PGPR properties among twelve isolates obtained from the rhizosphere of rice, Jerusalem artichoke, and vegetables.

IAA production was a key focus, and it was discovered that all twelve isolates produced IAA. Isolate S1-7 exhibited particularly high IAA content, reaching 74.43 μg/ml. Then, the crude extract of IAA was checked and confirmed by TLC method compared with IAA standard, the result presented that the R_f_ value of all of samples (from 12 isolates) was nearly at the same value of IAA standard, this seems to be that 12 isolates were capable to produce IAA (Fig. 1B). Many reported showed that 80% of the microorganisms isolated from the rhizosphere with various crops were showed the ability to synthesize and release auxins as secondary metabolite [33], such as *Enterobacter roggenkampii* ED5 produced 732.93 μg ml^−1^ IAA [34], *Pseudomonas aeruginosa* AL2-14B produced 79 μg ml^−1^ IAA [35]. The variability in IAA production among different bacterial species and strains is influenced by multiple factors, including culture conditions, growth stage, and substrate availability [36]. IAA is a well-known plant growth hormone that plays an important function in root formation. It has been shown to increase root surface area and length, giving plants better access to soil nutrients. Additionally, root exudation stimulated by IAA may create a favorable environment for rhizosphere bacteria by providing additional nutrients. However, the effect of IAA on plant growth is concentration dependent. For instance, Mirheidari [37] demonstrated that the application of 400 μg/ml IAA significantly increased roselle plant height compared to untreated controls, while Hu [38] found that 1.75 mg/l IAA enhanced maize growth. Conversely, excessive concentrations of IAA, such as 87.59 mg/l, led to a significant reduction in plant height and biomass, with even higher concentrations (175.18 and 437.95 mg/l) causing a marked decline in root biomass. Therefore, it is needed to determine the optimal IAA concentration produced by PGPR and its interaction with specific plant will be crucial for developing effective microbial inoculants such as biofertilizer.

Moreover, nitrogen fixation is one of the important abilities of PGPR, all 12 isolates could fix the nitrogen gas from atmosphere. The atmospheric N_2_ gas is converted into plant-utilizable forms by biological N_2_ fixation which changed nitrogen to ammonia and nitrate by nitrogen fixing microorganisms using a complex enzyme system which known as nitrogenase [39]. Both isolates (P8 and R15) showed the potential of phosphate solubilizing activity by expressing the solubilization index (SI) at 4.67 and 2.12 respectively. In natural, 95–99% of phosphorus presents in insoluble, immobilized and precipitated forms [40]; therefore, it is difficult for plants to absorb it [41] and mineralization of phosphorus by phosphate-solubilizing bacteria is an important trait which enhances the available forms of phosphorus in soils. The solubilization phosphorus activity in soils occurred via microorganisms by releasing organic acids or enzyme [42]. While only isolates P8 showed a value of 2.54 for the potassium solubilizing index, the ability of PGPR to solubilize potassium rock ií achieved through the production and secretion of organic acids, which has been widely investigated. In addition, in the report of Zang [43] also presented bacterial was screened from cassava, isolate A02 showed an abilities of nitrogen fixation, auxin (IAA) and solubilization of phosphate and it had the potential to be a good candidate strain for promoting crop yield.

Siderophore production, a key PGPR trait, was observed in eight isolates. This activity is normally revealed in soil, where siderophore released by microbes may bind to iron in soil for plant growth through different mechanisms, for instance, chelate and release of iron, direct uptake of siderophore-Fe complexes, or ligand exchange reaction. Siderophilic bacteria have been shown to play vital role in preventing diseases and enhancing the growth of plant [44]. Under saline stress conditions, siderophore-producing PGPR contributes to plant resilience by facilitating iron uptake, which is often limited in saline soils due to ionic imbalances and reduced bioavailability. In phytoremediation, siderophore-producing PGPR improves iron acquisition by plants, thereby enhancing their metabolic activities and stress tolerance. Iron is an essential micronutrient for both plants and microorganisms, playing a pivotal role in enzymatic activities, electron transport, and overall metabolic processes. Previous studies have demonstrated the effectiveness of siderophores in promoting plant growth under stress conditions. Vansuyt [45], it was found that the Fe-proverdine complex was synthesized by *Pseudomonas fluorescens* C7 and taken up by *Arabidopsis thaliana* plants, leading to an increase of iron inside plant tissues and to improve plant growth. Moreover, under 200mM salt stress, *Bacillus aryabhattai* has proven the ability to create a considerable amount of siderophore and then give the needed iron to the plants facing salinity stress [46]. Iron is essential for a variety of physiological and metabolic reactions in plants. It is a catalyst in enzymatic processes like photosynthesis, oxygen metabolism, the citric acid cycle, and the creation of DNA and RNA [47]. The capacity of PGPR to promote iron availability under saline conditions indicates that siderophore synthesis by isolates in this present study may play a vital role in crop yield improvement, particularly in tough situations.

In addition, this research showed a positive result for ACC deaminase production in 5 isolates. PGPR produces the ACC deaminase to facilitate better plants growth under various stress conditions. The interaction between root colonized by PGPR with ACC deaminase activity showed that plants exhibit greater tolerance against environmental stresses [48]. The enzymatic activity of ACC deaminase leads to the breakdown of ACC into α-ketobutyrate and ammonia. Reducing ACC levels prevents excessive ethylene accumulation in plant tissues under stress conditions, making it one of the most effective strategies for inducing salt stress tolerance [49]. Ethylene is a critical plant hormone that, at high concentrations, can inhibit root growth and overall plant development under stress conditions. By mitigating ethylene production, ACC deaminase-producing PGPR help sustain root elongation, enhance nutrient uptake, and improve plant biomass under saline conditions. The report of Nadeem [50] revealed that *E. aerogenes*, *Pseudomonas syringae* and *P. fluorescens* were produced ACC deaminase, which could induce salt tolerance by regulating of K/Na ratios, chlorophyll and proline level in maize. For bio-film production, isolates MSE-5 and S1-7 showed a positive result, biofilm formation is a suitable substance which was also showed the property in enhancing on the tolerance of plant in saline soil. The production of biofilms helps improve root colonization, increase water retention, and facilitate the sustained release of beneficial metabolites, thereby reinforcing plant growth under salt stress conditions [51].

### Plant Growth Promotion by PGPR- Biofertilizer

**Fig. 9:**
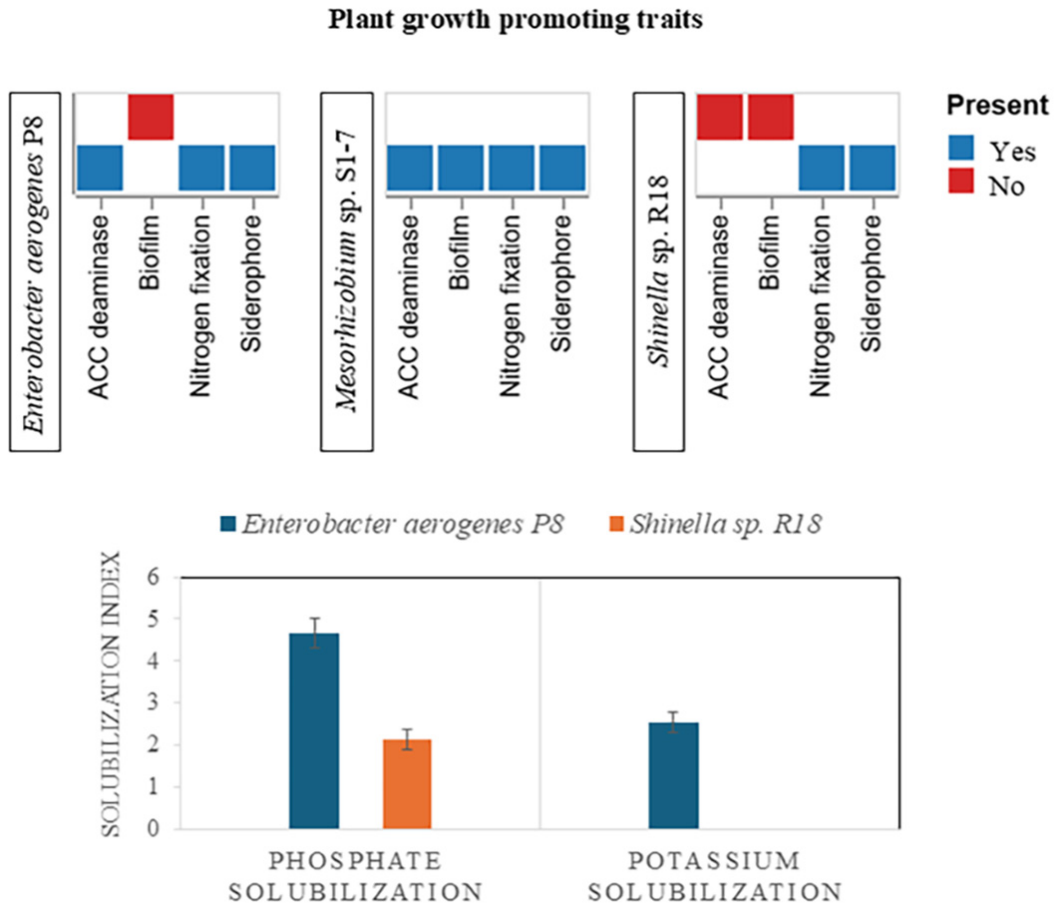
Plant Growth-Promoting Traits in *Enterobacter aerogenes* P8, *Shinella* sp. R18, and *Mesorhizobium* sp. S1-7.

In pot experiments, three isolates—P8 (*E. aerogenes*), R18 (*Shinella* sp.), and S1-7 (*Mesorhizobium* sp.)—were selected for biofertilizer production based on their high PGPR potential (Fig. 9) Several studies have highlighted the effectiveness of PGPRs as biofertilizers. For example, Kaur [52] reported co-inoculation of *Mesorhizobium* sp.(LGR -33) with *Pseudomonas* sp. PGPR 3 could enhance the yield of chickpea by 7.0% (desi) and 5.3% (kabuli) greater than when it used *Mesorhizobium* sp. alone. Similarly, Verma [53] also presented the result of nodulation formation (62 and 86%), dry weight of root (44 and 57%) and shoot (26 and 45%) were significantly recorded in co-inoculation of *Mesorhizobium* sp. and *Pseudomonas aeruginosa* greater than in the un-inoculated control under pot and field condition. In addition, another report presented that *Enterobacter* sp. UYSO10 and *Shinella* sp. UYSO24 confirmed the efficiency of PGP that affected the commercial sugarcane [54]. Sarin and Riddech [55] studied plant growth promoting properties of three isolates of rhizobacteria (*E. aerogenes* P8, *Bacillus tequilensis* N15, *Pseudomonas azotoformans* I2.1) which immobilized on rice husk ash carrier, this carrier could support the highest growth of these three kinds of bacteria and promoted growth of tomato plants in two different soils (normal and saline soil) conditions.

Under various salinity levels, SEM observations confirmed that rhizobacteria colonized rice root surfaces successfully. Our result was similar to the study of Cakmakci [56] which found that *Bacillus* and *Pseudomonas* spp. were an effective colonizer on wheat roots. The majority of the PGPR in plant colonized the root surface and thrived in spaces between root hairs [57]. Root exudates are an integral part of rhizosphere signaling events and regulate communication in beneficial plant–microbe interactions. Phenols, flavonoids, and organic acids secreted by roots, and it had been known as chemical signals for bacterial chemotaxis, quorum sensing and biofilm formation during rhizosphere colonization [58]. Plant root exudates are important factors for setting up on the structure of rhizobacterial community. It was well established that root exudates provided nutrients leading to enhancement of bacteria. The density of isolates *E. aerogenes* P8 and *Mesorhizobium* sp. S1-7 were not different in all of conditions. Notably, isolate *Shanell* sp. R18 showed reduced cell density under high salinity, emphasizing the need for tailored PGPR applications under specific stress conditions. These beneficial microbes were not only promoting plant growth. It could also protect plants from pathogens [59]. Moreover, it also increased the tolerance property in plant to abiotic stresses such as salinity, drought, nutrient deficiency [60]. The development of rhizosphere soil microbial communities is inextricably connected to the host plant and root exudates. Research has found that microbial populations in the rhizosphere differ greatly between plant species. PGPR interactions with plants have been commercialized and applied in scientific research for sustainable agriculture.

### Biofertilizer Production and Storage

The production methods for producing bio-fertilizer encompass various forms such as cell suspension, fermented with substrates prior to use, and immobilization on carriers. Shelf life and the release of rhizobacteria from bio-fertilizer were also tested after storage for one month. The suspension of rhizobacterial cells demonstrated higher cell survival rates compared to bacterial immobilization using sodium alginate form (granular bio-fertilizer) at both 4°C and room temperature. It is known that at 4°C, microbial cell activity and metabolism occur slowly, resembling a resting cell stage. In contrast, at room temperature, microbial cells remain in active form. The substrate in the granular form of bio-fertilizer was rapidly degraded by the active form of the microbes. Thus, the content of nutrients in granular may decrease and the pH value in the granular may shift towards acidic or alkali condition. This change could be as the un-favorite condition for growing microbes. Therefore, the density of microbes in granular form was decreased when stored at room temperature. Similar to the study of He *et al*. [61], they presented that *Raoultella planticola* Rs-2 was encapsulated in the form of capsules using sodium bentonite and sodium alginate (NaAlg) as a bio-fertilizer. The release efficacy from these two types of capsules occurred slowly and gradually increased. These microcapsules were spherical in shape, and their encapsulation efficiency was nearly 100%. In our experiment, microbial releasing was detected in soil under normal and salinity conditions. The results showed that on the 30^th^ day of incubation, the release of microbes in granular form (immobilized microbial cells in sodium alginate) was more effective than that from bacterial suspension. This meant that the carrier (in this study used sodium alginate) could protect the microbial cell from the abiotic and biotic stresses present in the environment. The suitable carrier for immobilizing microbial cells must be nontoxic, rich in nutrients and have the suitable content of water holding capacity.

Trivedi *et al*. [62] also found that *Bacillus subtilis* and *Pseudomonas corrugate* immobilized in sodium alginate beads, skim milk and charcoal exhibited effective enhancement in root and shoot length, as well as dry weight of maize when applied in cooler regions. In the report of Hassan and Bano [63], *Pseudomonas moraviensis* and *Bacillus cereus*, were used to produce bio-inoculants, employing wheat, maize straw and bagasse as carriers. The result showed that *P. moraviensis* and *B. cereus* had better survival efficiency in the carriers. Furthermore, the co-inoculation of these PGPRs in these carrier materials significantly reduced electrical conductivity (EC) and Na^+^ content in soil. The chlorophyll, protein, sugar, phytohormone, and antioxidant activities in leaves increased in content. Our data suggest that directly inoculating microbial cells (in the form of cell suspension) into natural soil may not be as effective for maintaining microbial survival compared to immobilization.

### Effect of Biofertilizers on Rice Seedlings in a Pot Experiment

Rice seedlings inoculated with biofertilizers exhibited significant growth enhancements under both normal and saline soil conditions compared to control treatments. PGPR inoculants showed the abilities of the promotion of several important agronomic plants, such as rice, maize, potato, bean, strawberry, cucumber and tomato. Kumar *et al*. [64] presented that the growth, yield and micronutrient in soil of wheat crops were enhanced by various combinations of microbial strains under pot and field experiments. These strains showed the abilities of PGPR such as nitrogen fixation, phosphate solubilization, HCN production, and IAA production. They also found that the triple combination of strains *B. megaterium*, *A. chlorophenolicus* and *Enterobacter* significantly increased plant height (17.5%), grain yield (79.8%), straw yield (78.6%), and biomass (26.7%) under pot conditions, suggesting the potential for an efficient microbial consortium for wheat production. In addition, our data showed that the growth of rice seeding in saline soil decreased compared to the growth in normal soil. However, the results showed that bio-fertilizer inoculation could promote the growth of rice more effectively than treatments without microbial inoculation.

Our result was similar to the study of Mendoza *et al*. [65], the bio-fertilizer of the strain *Bacillus* sp. XT13 and XT14 were produced by immobilizing on sodium alginate macro-beads, which showed efficacy in promoting guinea grass by responding on the stress under drought condition. The co-inoculants could enhance total dry biomass production by 94.4%. Co-inoculants also showed a significant effect on the antioxidant response in plants by decreasing on ascorbate peroxidase activity to 5.46%. In contrast, proline accumulation increased in plants when inoculated with the alginate macro-beads. Chen *et al*. [66] presented that *Bacillus amyloliquefaciens* (SQR9) influenced salt tolerance of maize by enhancing the content of chlorophyll product and stimulating the production of sugars and antioxidants within the tissues of the maize plant. Tirry *et al*. [67] reported on four selected potential PGPR strains (*Pseudomonas putida*, *Alcaligenes* sp., *Klebsiella* sp., and *Pseudomonas cedrina*.) and selected for inoculation on *M. sativa* on plants. It was found that the four strains allowed overcoming the negative effects of stress due to NaCl and increased in plant growth, occurred the root AMF colonization and gained in the content of leaves chlorophyll. Salt-tolerant species had higher chlorophyll content, whereas salt-sensitive species had lower levels [68]. However, the bacterial treatment did not significantly alter the chlorophyll content in rice leaves compared to the control in this study, indicated that while salinity stress typically causes chlorophyll degradation due to the oxidate damage and nutrient imbalances [69], the applied biofertilizer did not substantially mitigate or exacerbate these effects. It could be because the applied microbial inoculant did not reduce oxidative stress to a level that would significantly impact chlorophyll retention. Proline is a key osmoprotectant that accumulates in plant tissues during salt stress, protecting cellular structures and maintaining water balance [70]. It also acts as a protein-compatible molecule and a hydroxyl radical scavenger, protecting cellular redox potential during environmental stress [71, 72]. In this investigation, Proline buildup in rice was substantially higher under salty soil conditions than in normal soil, with the greatest amounts found in the saline control treatment. Proline protects cellular membranes and proteins against stresses such as high inorganic ion concentrations and harsh temperatures. This shows that the bio-fertilizers helped reduce salt stress, hence reducing the demand for proline accumulation. PGPR strains, particularly those that produce ACC deaminase, can reduce ethylene levels while increasing stress tolerance, lessening the plant's dependency on proline for osmotic adjustment. FDA, a microbial activity indicator, was significantly greater in all biofertilizer-inoculated treatments, indicating a strong microbial presence in the soil. The bacterial treatments raised FDA levels in saline soil when compared to the negative control, indicating that the introduced microbial inoculants enhanced microbial activity in the rhizosphere. Higher FDA hydrolysis levels suggest higher microbial colonization, nutrient cycling, and enzymatic activity in the soil, all of which can benefit to plant health and resilience under stress. These observed trends suggest that PGPR trains *E. aerogenes*, *Shinella* sp., and *Mesorhizobium* sp. can help plants cope with salinity stress through mechanisms other than direct chlorophyll stabilization, such as improving nutrient uptake, enhancing root growth, and modifying stress signaling pathways. Overall, \this study emphasizes the ability of PGPR-based biofertilizers to improve plant growth and resistance in harsh situations. Both liquid and granular formulations performed well, proving their suitability for a variety of agricultural applications. This finding is consistent with Sakpirom [73] who found that both liquid and solid biofertilizers obtained from agricultural waste greatly increased rice growth in paddy soils. Similarly, our research found that biofertilizers in alginate-based granular and liquid suspension forms significantly improved rice growth. However, while the granular and suspension form did not show significant differences in plant growth parameter and biomass accumulation in pot experiment, the granular form demonstrated a clear benefit in reducing proline accumulation under saline conditions, suggesting a stronger role in mitigating salt stress responses. Additionally, after 30 days, the granular form maintained a higher bacterial population in saline soil over time. This suggests that immobilized bacteria in granular form have better persistence in the soil environment, potentially long-term benefits. Moreover, the present study suggested that while short-term storage at room temperature (up to 1 month) is feasible, storing the biofertilizer at 4°C is preferable for maintaining higher microbial viability over an extended period. The FDA also emphasized the higher in the granular treatment, indicating enhanced microbial activity and soil functionality. The granular form has the major practical advantage of being reusable in succeeding harvests since the structure remains intact and the bacteria within is still active. While both formulations efficiently supported plant growth, the longevity, maintained bacterial activity, and enhanced soil microbial function of the granular form give a significant advantage for future uses in sustainable agriculture. These findings once again demonstrated the viability and usefulness of PGPR-based biofertilizers in promoting sustainable agriculture, even in severe environmental situations.

## Conclusion

This study examined the effectiveness of two types of PGPR-based biofertilizers—bacterial suspension and bacterial immobilization on sodium alginate (granular form)—in promoting rice growth in both normal and saline soils. The results clearly demonstrated that immobilized microbial cells in granular form were more effective, resulting in a slow-release mechanism that provided continuous plant growth. This type is especially advantageous for saline soils, where it promotes controlled nutrient release and microbial survival under stress. Based on these findings, the study supports prioritizing the development of biofertilizers including active components from three rhizobacterial isolates (*E. aerogenes*, *Shinella* sp., and *Mesorhizobium* sp.). The granular form of these biofertilizers should be prioritized, since its slow-release properties make it excellent for difficult conditions, such as saline soils. Biofertilizers are a sustainable and environmentally friendly option that not only promotes plant growth but also improves soil structure and nutrient cycle. Future studies should conduct a more detailed assessment of microbial viability after plant harvest, including long-term bacterial survival and soil microbial activity, to provide deeper insights into the sustained effects of the granular formulation. Additionally, research should focus on optimizing these formulations for broader agricultural application, maximizing their potential under a variety of farming conditions.

## Figures and Tables

**Table 1:** Plant Growth-Promoting Traits of 12 Rhizobacterial Isolates.

Isolates	Nitrogen fixation	Phosphate solubilization (SI)	Potassium solubilization (SI)	Siderophore	ACC deaminase	Biofilm
AVR1	+	-	-	-	-	-
I1.1	+	-	-	-	-	-
I3.5	+	-	-	-	-	-
MSE5	+	-	-	+	-	+
NAS3-4	+	-	-	+	+	-
P8	+	4.67 ± 0.35	2.54 ± 0.14	+	+	-
R15	+	-	-	+	+	-
R18	+	2.12 ± 0.24	-	+	-	-
S1-7	+	-	-	+	+	+
S2-7	+	-	-	+	+	-
TYS2-1	+	-	-	-	-	-
TYS4-3	+	-	-	+	+	-

**Table 2:** Growth parameters of rice seedling at 30 days’ old.

Treatments	Shoot length (cm)	Root length (cm)	Leaf width (cm)	Number of leaves	Biomass (g of dry weight)
T1: Normal soil (N)	44.5 ± 1.91^cd^	33.8 ± 3.86	0.43 ± 0.05^bc^	4.0 ± 0.00^b^	0.94 ± 0.31^bcd^
T2: Saline soil (S)	42.0 ± 2.16^d^	36.0 ± 3.56	0.37 ± 0.05^c^	2.6 ± 0.50^c^	0.67 ± 0.14^d^
T3: Bacterial in alginate (N)	49.8 ± 1.71^ab^	24.8 ± 2.06	0.50 ± 0.08^ab^	4.0 ± 0.00^b^	1.64 ± 0.28^a^
T4: Bacterial in alginate (S)	46.5 ± 0.58^bcd^	33.8 ± 4.57	0.47 ± 0.00^ab^	3.0 ± 0.00^c^	1.18 ± 0.25^bc^
T5: EM (N)	47.3 ± 4.27^abc^	37.0 ± 3.16	0.45 ± 0.05^ab^	4.3 ± 0.50^ab^	1.18 ± 0.27^bc^
T6: EM (S)	46.8 ± 3.30^bcd^	31.3 ± 6.34	0.44 ± 0.06^abc^	3.0 ± 0.00^c^	0.93 ± 0.14^cd^
T7: Bacterial suspension (N)	51.8 ± 5.68^a^	32.3 ± 3.40	0.53 ± 0.05^a^	4.5 ± 0.58^a^	1.34 ± 0.26^ab^
T8: Bacterial suspension (S)	47.8 ± 4.11^abc^	29.5 ± 2.52	0.44 ± 0.06^abc^	3.0 ± 0.00^c^	1.33 ± 0.26^abc^
F-test	*	ns	*	**	**
%CV	7.13	20.85	11.68	9.06	14.17

S: Saline soil that adjusted with NaCl (0.5% w/w), N: Normal soil

ns: non-significant different *Significant different at 95% (*p* < 0.05) ** Significant different at 95% (*p* < 0.01).

Different letters in the column represented significant differences among treatments (*p* < 0.05) according to the LSD test.

**Table 3:** pH and electrical conductivity value in soils.

Soils	pH	Electrical conductivity (dS/m)
Original soil	7.18-7.37	0.28-0.49
Normal soil (harvest)	7.25-7.47	0.34-0.56
Saline soil (harvest)	7.02-7.14	3.98-4.56
